# Lung inflammation and interstitial fibrosis by targeted alveolar epithelial type I cell death

**DOI:** 10.3389/fimmu.2023.1261483

**Published:** 2023-09-28

**Authors:** Sandra Carignon, Dorian De Moura Rodrigues, David Gosset, Elodie Culerier, Sarah Huot-Marchand, Florence Savigny, Eric Kaya, Valerie Quesniaux, Aurélie Gombault, Isabelle Couillin, Bernhard Ryffel, Marc Le Bert, Nicolas Riteau

**Affiliations:** ^1^ University of Orleans and CNRS, Immunologie et Neurogénétique Expérimentales et Moléculaires -UMR7355, Orleans, France; ^2^ Center for Molecular Biophysics, CNRS Unité propre de recherche 4301, Orleans, France

**Keywords:** AT1 cell depletion, sterile inflammation, lung injury, iDTR, cell death

## Abstract

**Introduction:**

The pathogenesis of chronic lung diseases is multifaceted with a major role of recurrent micro-injuries of the epithelium. While several reports clearly indicated a prominent role for surfactant-producing alveolar epithelial type 2 (AT2) cells, the contribution of gas exchange-permissive alveolar epithelial type 1 (AT1) cells has not been addressed yet. Here, we investigated whether repeated injury of AT1 cells leads to inflammation and interstitial fibrosis.

**Methods:**

We chose an inducible model of AT1 cell depletion following local diphtheria toxin (DT) administration using an iDTR flox/flox (idTR^fl/fl^) X Aquaporin 5CRE (Aqp5^CRE^) transgenic mouse strain.

**Results:**

We investigated repeated doses and intervals of DT to induce cell death of AT1 cells causing inflammation and interstitial fibrosis. We found that repeated DT administrations at 1ng in iDTR^fl/fl^ X Aqp5^CRE^ mice cause AT1 cell death leading to inflammation, increased tissue repair markers and interstitial pulmonary fibrosis.

**Discussion:**

Together, we demonstrate that depletion of AT1 cells using repeated injury represents a novel approach to investigate chronic lung inflammatory diseases and to identify new therapeutic targets.

## Introduction

Repeated respiratory epithelial barrier injury contributes to the pathogenesis of several chronic lung diseases including interstitial lung diseases (ILD) and emphysema, a characteristic of chronic obstructive pulmonary disease (COPD). Idiopathic pulmonary fibrosis (IPF) is the most severe type of ILD characterized by dysregulated alveolar repair leading to pathological lung scarring (1–3). Recent developments in the field clearly establish that IPF pathophysiology relies on recurrent micro-injuries and alveolar epithelial damage leading to cell death, tissue remodeling and a maladaptive repair response (4, 5). COPD, the third leading cause of death worldwide, is a disease state usually characterized by progressive airway limitation and punctuated by life-threatening clinical exacerbations mainly elicited by bacterial and viral infections (6).

The importance of lung epithelial damage in COPD pathogenesis has been recently reviewed (7). While being two distinct diseases, lung aging and loss of epithelial integrity are major features seen in both COPD and IPF (3). The alveolus is lined with very thin alveolar epithelial type I (AT1) cells allowing passive gas diffusion and alveolar epithelial type 2 (AT2) cells which support lung function by secreting surfactant proteins. AT2 cell dysfunction has been implicated as an important driver of lung fibrosis in IPF (8); however other cell types are likely to be involved in lung disease including immune cells (9). Importantly, AT2 cells also display stem cell properties and contribute to AT1 cell renewal during homoeostasis as well as following lung injury (10).

IPF patients display significant loss of AT1 cells; however the specific contribution of AT1 cells to disease development has not been evaluated (2). Here, we focused on the role of AT1 cells lining the alveoli, which represent the largest surface of the lung exposed to air. Instead of using pollutant or bleomycin which cause damage to a broad range of cells, we used a genetic model of inducible and progressive AT1 cell death to explore the development of inflammation and interstitial lung fibrosis. For that purpose, we generated a cell-specific depletion model in mice using the CRE-inducible diphtheria toxin receptor (DTR) transgenic mice (iDTR) system (11).

Briefly, DTR gene expression, under control of the ubiquitous ROSA promotor, is blocked by a STOP cassette flanked by loxP sequences. In selected cell types, CRE recombinase activity removes the STOP cassette leading to DTR expression allowing these cells to become sensitive to diphtheria toxin (DT). DT binds to surface expressed DTR allowing its internalization and release of the catalytic A subunit of DT which selectively inactivates ADP-ribosylating ribosomal elongation factor-2 (EF-2), thus dampening protein synthesis and inducing cell death. DT is a very potent toxin and an early study demonstrated that a single internalized DT molecule is enough to trigger cell death (12).

Here, we investigated the effect of alveolar integrity disruption by ablation of AT1 cells using the DT inducible AQP5 CRE x iDTR floxROSA stop mouse (11). Our results show that AT1 cell ablation is associated with low grade inflammation, increased repair and remodeling factors with progression to alveolar septal fibrosis. This may present a novel model of repeated epithelial injury leading to chronic lung inflammatory diseases such as IPF and a potential tool to test drug candidates.

## Materials and methods

### Mice

We crossed aquaporin 5 CRE mice (Aqp5^CRE^: Aqp5tm1.1(cre)Pfl)(13) with iDTR flox (Gt(ROSA)26Sortm1(HBEGF)Awai)(11) mice (iDTR^fl/fl^) to ablate lung epithelial AT1 cells upon diphtheria toxin (DT) treatment. iDTR^fl/fl^ mice were a gift from Ary Waisman (IMB Mainz, Germany). Control wild-type C57BL/6J (WT) mice were purchased from Janvier Laboratory, France.

Both Aqp5^CRE^ and iDTR^fl/fl^ strains were generated on a 129 background and backcrossed >10 times to a C57BL/6 background. Breeding was performed by crossing iDTR^fl/fl^ Aqp5^WT^ with iDTR^fl/fl^ Aqp5^CRE^ mice and littermates were used for experiments. Mice were housed in our specific pathogen-free animal facility at CNRS (TAAM UPS44, Orleans, France). For experiments, adult (8–14-week-old) males or females were transferred to the experimental animal facility and monitored daily. Of note, in the original publication of directed CRE expression in AT1 cells, the authors could exclude that CRE activity was also occurring in some AT2 cells since a 129/C57BL/6 background was used (13).

### Ethics

All animal experiments complied with the French Government animal experiment regulations and were approved under APAFIS#21753. Clinical score was determined daily based on mice appearance and behavior. Appearance was determined based on standard parameters including eye, fur and ear monitoring (https://www.nc3rs.org.uk/grimacescales) and behavior monitoring included mobility, posture and social interaction.

### Treatments

Diphteria Toxin (DT) from *Corynebacterium diphtheriae* (1 and 10 ng per mouse, Merck, #322326) in saline or saline alone was administered into the airways by oropharyngeal administration in a volume of 40 μl under light isoflurane anesthesia. The schedules, doses and durations of the different experiments varied as DT at 10 ng dose had important off-target effects, and repeated DT 1ng doses caused major weight loss requiring early analyses. Schematic outlines of the different protocols are provided with the figures in the main manuscript.

### Bronchoalveolar lavage and cell count

Mice were euthanized by progressive CO_2_ exposure using a Prodigy Lab Control Unit (Smartbox) and bronchoalveolar lavage fluid (BALF) was performed as previously described (14). Differential cell counts were routinely counted on cytospin preparations (Cytospin 3, Thermo Shandon) after May-Grunewald Giemsa staining (Sigma Aldrich, St Louis, MO) according to the manufacturer’s instructions and at least 200 cells were counted using standard morphological criteria.

### Lung homogenization

After BALF collection, lungs were perfused with ISOTON (Beckman Coulter) to flush the vascular compartment. The lung inferior and post-caval lobes were processed using a Precellys tissue homogenizer (Bertin Instruments) in 1 ml phosphate-buffered saline (PBS) with complete Protease Inhibitor Cocktail (Roche). The extracts were centrifuged for 10 min at 9,000 g and supernatants stored at -80°C for ELISA or western blot assays.

### Lung histology

Lung left lobes were fixed in 4% buffered formaldehyde and paraffin-embedded under standard conditions. Lung sections (3 μm) were stained with hematoxylin and eosin (H&E) or Sirius red/Fast green. The slides were scanned using NanoZoomer (Hamamatsu Photonics, France). Scoring of inflammation, remodeling and fibrosis was assessed by two independent reviewers using a semi-quantitative score with increased extent of the lesion (0-5) as described previously (15).

### Mediator measurement

BALF supernatants and lung homogenates were analyzed using ELISA assay kits for murine MMP-9 and TIMP-1 according to the manufacturer’s instructions (R&D system, Minneapolis, MN).

### Collagen assay

BALF collagen content was measured using the Sircol collagen dye binding assay (Biocolor Ltd., Northern Ireland) according to the manufacturer’s instructions.

### Double-stranded (ds) DNA quantification

Cell-free dsDNA was measured in the BALF fluid using Quant-iT PicoGreen dsDNA reagent (Invitrogen, Carlsbad, CA), according to the manufacturer’s protocol.

### Quantitative PCR

RNA was purified from lung homogenates using Tri-Reagent (Sigma-Aldrich, Saint-Louis, MO) extraction protocol. RNA reverse transcription into cDNA was carried out with GoTaq qPCR-Master Mix (Promega, Madison, WI). RT-qPCR was performed with Fast SYBR Green Master mix (Promega) on an ARIA MX (Agilent Technologies, Santa Clara, CA). Primers for *Fn1* (#QT00135758), *Col3a* (#QT01055516) and *Timp1* (#QT00996282) were purchased from Qiagen (Qiagen, Hilden, Germany). RNA expression was normalized to *Gapdh* (#QT01658692, Qiagen, Hilden, Germany) expression and analyzed using the ΔΔCt method.

### Flow cytometry

Lungs were cut into small pieces and digested using a 1 mg/ml DNase (DN25, Sigma Aldrich, St Louis, MO)/125µg/ml collagenase (Liberase™, Sigma Aldrich, St Louis, MO) solution for 45 min at 37°C and filtered using a 40 µm cell strainer. BALF and lung single cell suspensions were incubated for 20 min at RT with Fc block (ThermoFisher Scientific, Waltham, MA), washed and stained with surface markers including a Fixable Viability Dye eFluor 780 (ThermoFisher Scientific, Waltham, MA). Cells were fixed and permeabilized using a Fixation/Permeabilization kit (ThermoFisher Scientific, Waltham, MA) and stained with intracellular markers. Antibody clones and dilutions used are detailed in **Supplementary Table 1**. All samples were acquired on an LSR Fortessa X20 flow cytometer (BD Biosciences, San Jose, CA) and analyzed using FlowJo software (TreeStar).

### Western analysis

20 μg of proteins (Pierce BCA protein assay, ThermoFisher Scientific, Waltham, MA) were denatured by boiling (95°C, 5 min) in reducing SDS sample buffer, separated by SDS-PAGE and transferred to nitrocellulose membranes (GE Healthcare Life Sciences, Amersham, UK). After blocking in 5% Blotting-Grade Blocker (BioRad, France) and washing in Tris-Buffered saline (TBS)-0.1% Tween^®^ 20, membranes were incubated with primary mouse [anti-STING] antibody. Membranes were washed and incubated with relevant secondary antibody conjugated to horseradish peroxidase (HRP) for two hours at RT. Anti-actin antibody was HRP-conjugated (Sigma Aldrich, ref A3854). Detection was performed with ECL Western-blotting Detection Reagent (GE Healthcare Life Sciences, Amersham, UK) and luminescence acquired using a Multi-application gel imaging system PXi software (Syngene). Band intensity was quantified using ImageJ (NIH, USA).

### Immunofluorescence

Lung tissues were fixed in 4% paraformaldehyde (PFA) (Sigma Aldrich, St Louis, MO) and then sequentially incubated at 4°C with 5%, 10% and 20% sucrose over a one-week period. Lungs were embedded in tissue-tek (OCT^®^) and stored at -80°C. 10 μm lung sections were cut on a cryostat (Leica, Solms, Germany) and heated at 80° C for 30 min in 10 mM citrate pH = 6. Lung cells were permeabilized with 0.5% Triton X-100, blocked in 1% BSA, 10% FCS, 0.1% Triton X-100 in PBS for 1h, washed three times in TBS and incubated overnight with primary antibodies (see **Supplementary Table 1** for details) in PBS containing 2% BSA, 10% FCS and 0.5% Triton X-100 at 4°C. Lung sections were washed with TBS and incubated with relevant secondary antibodies conjugated with Alexa Fluor dyes for 1h at RT. After washing, cells were counterstained with DAPI for 10 min at RT, washed with PBS and mounted onto microscope slides (mowiol). Slides were visualized using a Zeiss Axio Observer Z7 microscope coupled with LSM 980 AiryScan2 module device (Carl Zeiss Co. Ltd., Jena, Germany). Axio Observer Z7 microscope (Carl Zeiss Co. Ltd., Jena, Germany) inverted microscope was equipped with a Plan-Apochromat 20X objective (NA = 0.8). Images were acquired using ZEN v2.3 pro Zeiss software (Carl Zeiss Co. Ltd., Jena, Germany).

### Statistical analyses

Statistical tests of selected populations were performed using the non-parametric Kruskal-Wallis test. Body weight variation plots were analyzed using two-way ANOVA corrected for multiple comparisons employing Tuckey’s test. Results were considered significant at p <0.05.

## Results

### Generation of inducible AT1 cell deficient mice and model validation

**Figure 1:**
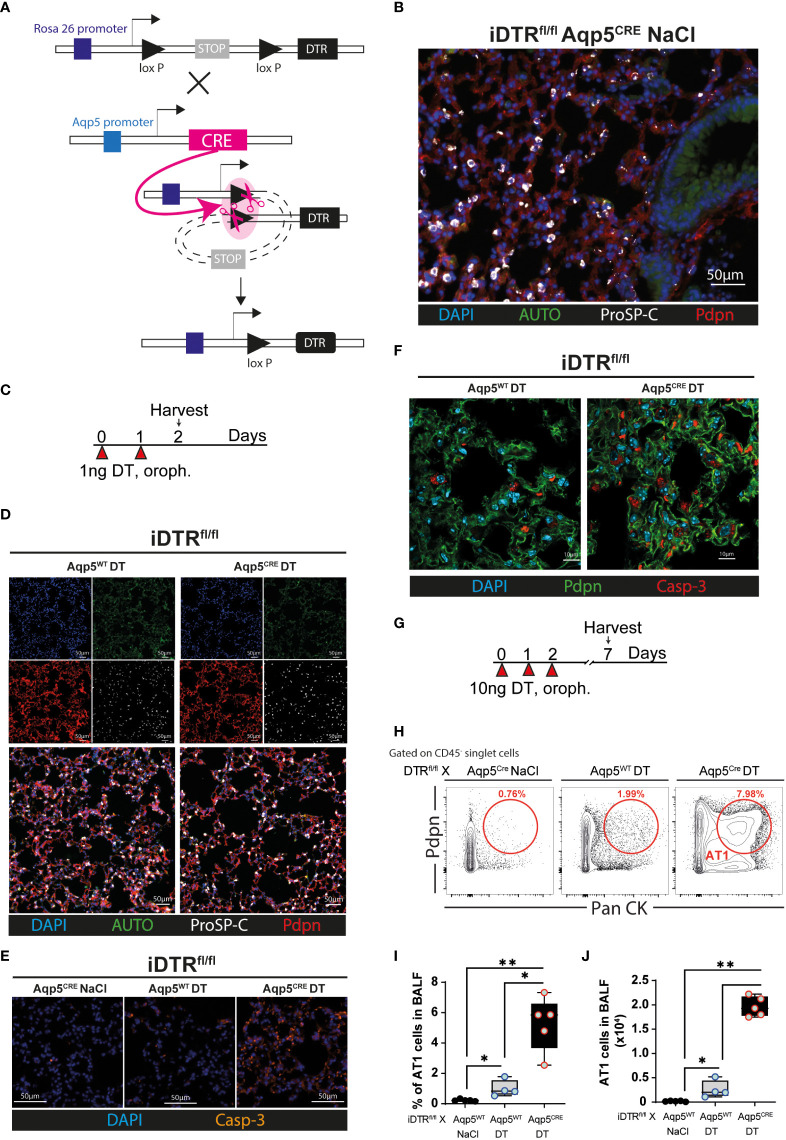
Generation and validation of iDTR^fl/fl^ x Aqp5^CRE^ mice. A CRE recombinase cassette was placed under control of the aquaporin5 (AQP5) promoter in a knockin mouse strain for ATI cell-specific targeting and crossed with iDTR floxROSA stop mice. In the absence of CRE recombinase activity, a stop cassette impedes DTR expression. In AT1 cells, *Aqp5* promoter activation induces CRE expression which removes the STOP cassette inserted upstream of the DTR gene and allows its transcription **(A)**. iDTR^fl/fl^ x *Aqp5* ^CRE^ mice were treated with NaCl for two consecutive days and lungs harvested 24 h after the last exposure and lung sections were stained with anti-podoplanin (Pdpn) and anti-prosurfactant protein C (ProSP-C) to visualize AT1 and AT2 cells, respectively **(B)**. iDTR^fl/fl^ x Aqp5^CRE^ mice and control iDTR^fl/fl^ x Aqp5^WT^ mice were treated daily for two days with 1 ng of DT in the airways by oropharyngeal administration and harvested 24 h after the last administration **(C)**. Lung immunofluorescence imaging suggests discrete alveolar loss in iDTRfl/fl x Aqp5CRE **(D)**, with strong increases of cleaved caspase-3 (Casp-3) staining in AT1 cells **(E, F)**. Mice were treated daily for three consecutive days with 10 ng of DT and harvested at day 7 **(G)**. Representative flow cytometry plot **(H)**, percentage **(I)** and total numbers **(J)** of AT1 cells recovered in the bronchoalveolar lavage fluid (BALF) gated as Pdpn^+^ and pancytokeratin (Pan CK)^+^. Each dot represents an individual sample; data are representative of 2 independent experiments, showed as mean ± SEM, *p < 0.05; **p < 0.01.

We crossed the iDTR floxROSA stop (iDTR^fl/fl^) mouse strain with aquaporin 5 (AQP5) CRE (Aqp5^CRE^) mice (**Figure 1A**). The AQP5 promoter drives CRE expression selectively in AT1 cells (13) where it removes a STOP cassette inserted upstream of the DTR gene allowing its transcription and cell death upon DT treatment (**Figure 1A**). We performed immunofluorescence imaging of lung tissue to visualize AT1 and AT2 cells. Saline treated-iDTR^fl/fl^ Aqp5^CRE^ showed normal alveolar distribution, with an extended surface covered by podoplanin (pdpn)-expressing AT1 cells and cuboidal prosurfactant protein C (proSP-C)-expressing AT2 cells (**Figure 1B**). Two days after low-dose DT treatment (**Figure 1C**), immunofluorescence of lung sections showed that iDTR^fl/fl^ Aqp5^CRE^ mice tend to display increased alveolar space (**Figure 1D**), but no significant differences were achieved. However, at this early time point a strong pro-apoptotic signature was observed in DT treated-iDTR^fl/fl^ Aqp5^CRE^ mice with high numbers of caspase-3 positive AT1 cells as compared to DT treated-iDTR^fl/fl^ Aqp5^WT^ mice (**Figures 1E, F** **)**. At this time point no AT1 cells were found in the bronchoalveolar lavage (BAL) fluid as an indication of epithelial cell loss (data not shown).

We then used a modified protocol, corresponding to three consecutive DT administrations at 10 ng per mouse with sacrifice five days later (**Figure 1G**). Flow cytometry on BAL cells was performed to quantify the AT1 cell population using a common epithelial cell marker (Pan-cytokeratin) and an AT1 cell-specific marker (Podoplanin). As expected, almost no AT1 cells were found in the BAL of iDTR^fl/fl^ Aqp5^CRE^ mice treated with saline and a slight AT1 increase was observed in control mice that received DT but do not express the CRE recombinase (iDTR^fl/fl^ Aqp5^WT^) (**Figures 1H–J**). In contrast, DT administration to iDTR^fl/fl^ Aqp5^CRE^ mice leads to a significant increase in AT1 cells (**Figures 1H–J**). Of note, non-AT1 epithelial cells (CD45^-^ PanCK^+^ Pdpn^-^) were also present, suggesting mild off-target effects since residual CRE expression in other cell types and especially cell death of some AT2 cells cannot be fully excluded. Other minor PanCK^+^ epithelial cell populations, including club cells and basal cells, might also be present. Together, these data show AT1 cell death upon DT treatment leading us investigate the inflammation and lung pathology associated with this phenotype at different time points.

### DT-induced AT1 cell depletion causes inflammatory cell recruitment and early lung inflammation

**Figure 2:**
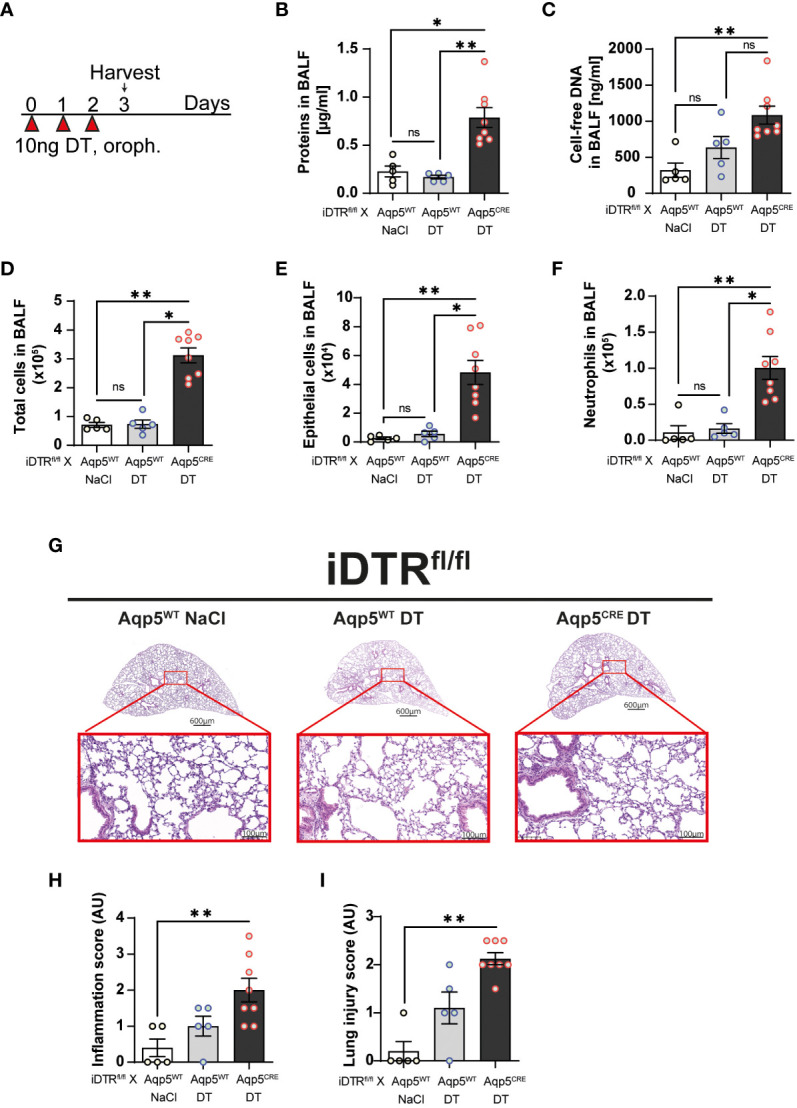
DT-induced AT1 cell ablation causes inflammatory cell recruitment and early lung inflammation at day 3. iDTR^fl/fl^ x Aqp5^CRE^ mice and control iDTR^fl/fl^ x Aqp5^WT^ mice were treated daily for three consecutive days with 10 ng of DT in the airways by oropharyngeal administration and harvested 24 h after the last administration. An additional iDTR^fl/fl^ x Aqp5^WT^ mice control group received saline (NaCl) administration **(A)**. Cell free protein **(B)** and cell free-DNA **(C)** contents in the BALF were significantly increased as well as total cell counts **(D)**, especially epithelial cells **(E)** and neutrophils **(F)** in the DT-treated iDTR^fl/fl^ x Aqp5^CRE^ group. Lung tissue was stained with hematoxylin and eosin (HE) **(G)** and lung inflammation and injured scored **(H, I)**. Each dot represents an individual sample; data are representative of a single experiment, shown as mean ± SEM, *p < 0.05; **p < 0.01.

Three daily oropharyngeal administration of 10 ng DT with analysis 24 hours after the last treatment (**Figure 2A**) causes disruption of the respiratory barrier with protein leak (**Figure 2B**) and increased cell-free DNA content in iDTR^fl/fl^ Aqp5^CRE^ mice (**Figure 2C**). This was accompanied by increased total cell number in the BAL (**Figure 2D**), especially epithelial cells (**Figure 2E**) and neutrophils (**Figure 2F**). Mild inflammatory foci with mononuclear cell infiltration are visible in the lung by microscopy (**Figure 2G**) and lung inflammation and injury were significantly increased in iDTR^fl/fl^ Aqp5^CRE^ mice (**Figures 2H, I** **)**. Of note, we found that DT administration to control iDTR^fl/fl^ Aqp5^WT^ mice (i.e. mice that do not express DTR in their AT1 cells) does not induce an increase in BAL epithelial cells or immune cell infiltration, however sparse cell infiltrates were detected by microscopy.

### Lung inflammation and fibrosis upon targeting AT1 cells 8 days after two DT treatments

**Figure 3:**
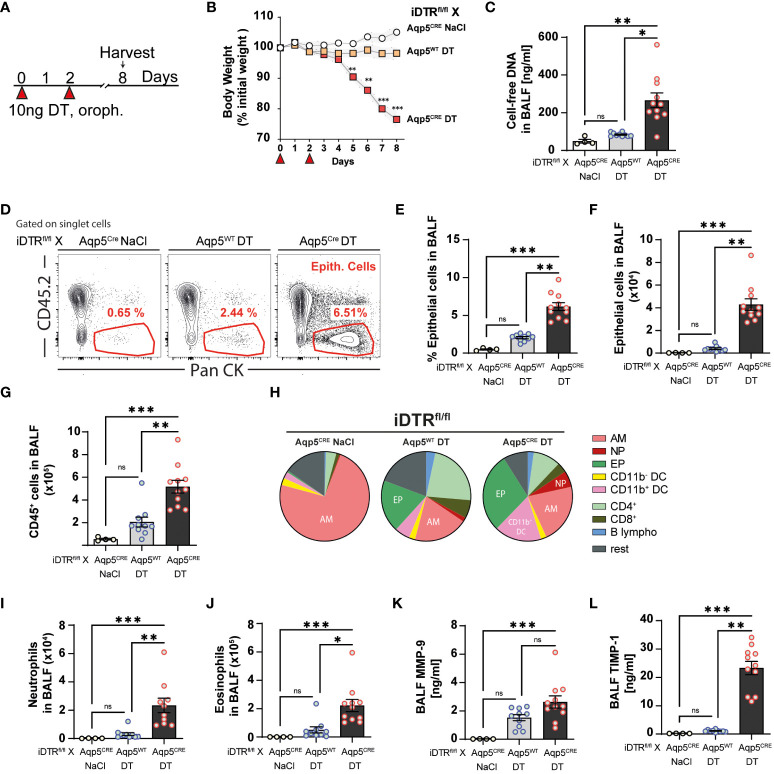
DT-induced AT1 cell ablation causes inflammatory cell recruitment at day 8. iDTR^fl/fl^ x Aqp5^CRE^ mice and control iDTR^fl/fl^ x Aqp5^WT^ mice were treated at days 0 and 2 with 10 ng of DT in the airways by oropharyngeal administration and harvested at day 8. An additional iDTR^fl/fl^ x Aqp5^CRE^ mice control group received saline administration **(A)**. Significant body weight loss was observed in DT-treated iDTR^fl/fl^ x Aqp5^CRE^ mice starting at day 5 **(B)**. Increased BALF cell-free self-DNA content in DT-treated iDTR^fl/fl^ x Aqp5^CRE^ **(C)**. Representative flow cytometry plots **(D)**, percentage **(E)** and total numbers **(F)** of BALF epithelial cells. Numbers of CD45^+^ cells in the BALF **(G)** and pie chart showing main immune cell populations **(H)**. Increased neutrophils **(I)** and eosinophils **(J)** in DT-treated iDTR^fl/fl^ x Aqp5^CRE^ mice as well as elevated levels of remodeling factors MMP-9 **(K)** and TIMP1 **(L)** in the BALF. Each dot represents an individual sample; data are representative of 2 independent experiments, showed as mean ± SEM, *p < 0.05; **p < 0.01; ***p < 0.01; ns, non-significant.

Since three daily DT administrations at 10 ng caused up to 20% loss of body weight, we decided to administer 10 ng only twice and investigate the effect of AT1 cell ablation at a later time point (**Figure 3A**). In contrast to the control group (DT-treated iDTR^fl/fl^ Aqp5^WT^ mice), DT administration to iDTR^fl/fl^ Aqp5^CRE^ mice leads to significant body weight loss starting at day 5 (**Figure 3B**). At day 8, increased cell-free DNA in the BAL was detected (**Figure 3C**), suggesting ongoing cell death. Indeed, AT1 cell depletion in DT-treated iDTR^fl/fl^ Aqp5^CRE^ mice results in a significant increase of epithelial cells in BAL (**Figures 3D–F**) reflecting epithelial damage and barrier injury, while DT-treated control iDTR^fl/fl^ Aqp5^WT^ mice showed minimal increase of BAL epithelial cells (**Figures 3D–F**). AT1 cell loss leads to increased immune cell recruitment in the BAL (**Figure 3G**), especially innate immune cells (**Figure 3H**) including neutrophils and eosinophils (**Figures 3I, J** **)**. Furthermore, MMP-9 and TIMP-1 levels in the BAL, markers of tissue remodeling and evolution towards fibrosis, were also significantly increased in mice that underwent DT-driven AT1 cell loss (**Figures 3K, L** **)**.

**Figure 4:**
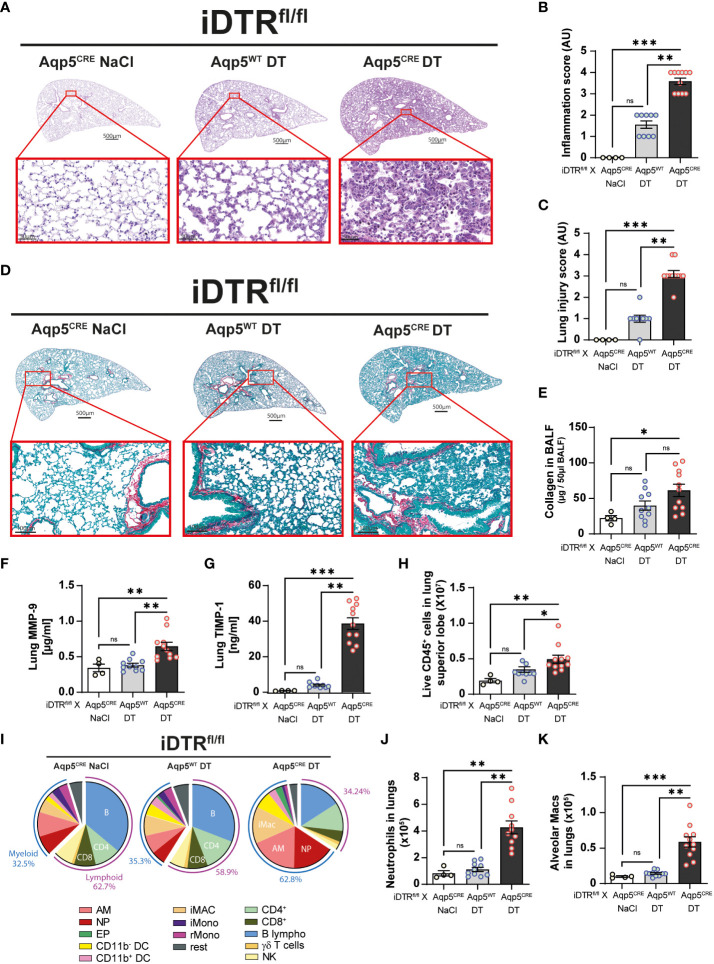
DT-induced AT1 cell ablation causes lung inflammation and early fibrosis at day 8. Mice were treated as in **Figure 3A** and lung tissue was stained with H&E **(A)** and scored for inflammation **(B)** and injury **(C)**. Collagen deposition in the lungs was assessed by Red Sirius staining showing collagen fibers in purple **(D)** and collagen content measured by Sircol assay **(E)**. Increased levels of remodeling factors MMP-9 **(F)** and TIMP1 **(G)** were found in the lungs. CD45^+^ cell numbers were increased in the lungs of DT-treated iDTR^fl/fl^ x Aqp5^CRE^ mice **(H)**. Main immune cell populations are showed in a pie chart **(I)** and significant increase of both neutrophils **(J)** and alveolar macrophages **(K)** were observed. Each dot represents an individual sample; data are representative of 2 independent experiments, showed as mean ± SEM, *p < 0.05; **p < 0.01; ***p < 0.001; ns, non-significant.

We next analyzed lung tissue and observed increased inflammation and epithelial injury in DT-treated iDTR^fl/fl^ Aqp5^CRE^ mice (**Figures 4A–C**) at day 8 after two DT doses at 10ng. Increased collagen deposition in the lungs assessed by Red Sirius staining (**Figure 4D**) and collagen content in the BAL were observed (**Figure 4E**) supporting interstitial fibrosis. Tissue remodeling factors MMP-9 and TIMP-1 levels in lungs were also significantly increased (**Figures 4F, G**) indicating ongoing fibrotic processes. This was accompanied by an increase of immune cells in the lungs (**Figure 4H**) and especially myeloid cells (**Figure 4I**), in particular neutrophils (**Figure 4J**) and alveolar macrophages (**Figure 4K**).

### Inflammation, repair and interstitial fibrosis upon repeated low dose DT instillation

**Figure 5:**
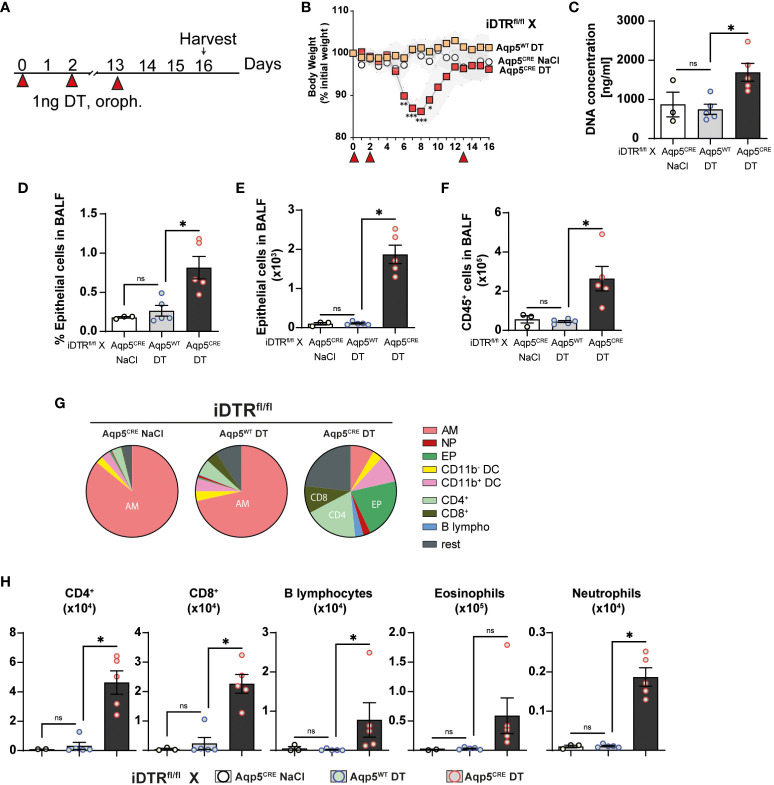
Low dose DT-induced AT1 cell ablation causes inflammation at day 16. iDTR^fl/fl^ x Aqp5^CRE^ mice and control iDTR^fl/fl^ x Aqp5^WT^ mice were treated at days 0, 2 and 13 with low dose (1ng) of DT in the airways by oropharyngeal administration and harvested at day 16. An additional iDTR^fl/fl^ x Aqp5^CRE^ mice control group received saline administration **(A)**. Significant body weight loss in DT-treated iDTR^fl/fl^ x Aqp5^CRE^ mice starting at day 6 with full recovery by day 12 **(B)**. Increased BALF cell-free self-DNA content in DT-treated iDTR^fl/fl^ x Aqp5^CRE^ **(C)**, together with increased percentage **(D)** and total numbers **(E)** of epithelial cells as well as CD45^+^ immune cells **(F)**. Pie chart showing main BALF immune cells populations **(G)** and increased numbers of eosinophils, CD4^+^ and CD8^+^ T lymphocytes in DT-treated iDTR^fl/fl^ x Aqp5^CRE^ mice and neutrophils and B lymphocytes in a lesser extent **(H)**. Each dot represents an individual sample; data are representative of 2 independent experiments, showed as mean ± SEM, *p < 0.05; ns, non-significant.

**Figure 6:**
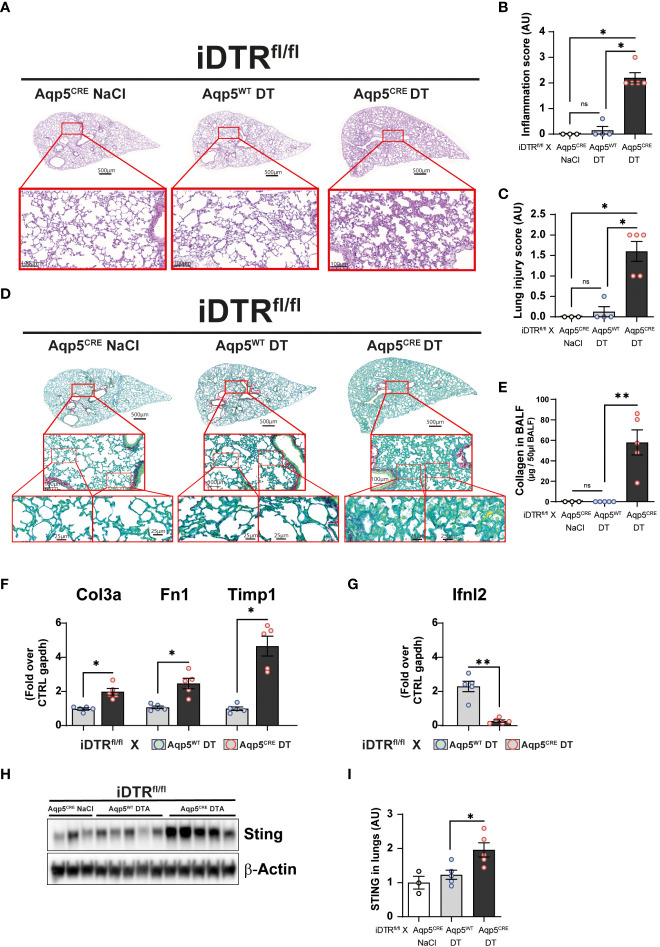
Low dose DT-induced AT1 cell ablation causes lung inflammation and fibrosis at day 16. Mice were treated as depicted in **Figure 5A** and lung tissue was stained with H&E **(A)** and scored for inflammation **(B)** and injury **(C)**. Collagen deposition in the lungs was assessed by Red Sirius stain **(D)** and collagen content in the BALF measured by Sircol assay **(E)**. Gene expression of fibrosis markers collagen (Col3a), fibronectin (Fn1) and Timp1 are increased in DT-treated iDTR^fl/fl^ x Aqp5^CRE^ mice **(F)**, whereas interferon lambda2 (Ifnl2) expression is strongly decreased **(G)**. Western blot assay showed increased STING expression in DT-treated iDTR^fl/fl^ x Aqp5^CRE^ mice **(H, I)**. Each dot represents an individual sample; data are representative of 2 independent experiments, showed as mean ± SEM, *p < 0.05; **p < 0.01; ns, non-significant.

We next investigated whether repeated low dose DT administrations (1 ng per mouse, given at days 0, 3 and 13) (**Figure 5A**) would lead to marked inflammation and interstitial fibrosis at day 16. Low dose DT to iDTR^fl/fl^ Aqp5^CRE^ mice leads to significant weight loss peaking at day 8 followed by a rapid and almost complete recovery (**Figure 5B**). Interestingly, we still noted increased free DNA in the BAL (**Figure 5C**) together with a significant percentage and number of epithelial cells in the BAL (**Figures 5D, E**). Increased immune cells were detected confirming previous data (**Figure 5F**); however, at this time point adaptive immune cells were also recruited (**Figures 5G, H**). Histological analyses of lung tissue demonstrated persistent inflammation and injury in DT-treated iDTR^fl/fl^ Aqp5^CRE^ mice at day 16 (**Figures 6A–C**) as well as increased collagen deposition in the lungs assessed by Red Sirius staining (**Figure 6D**) and collagen content in the BAL (**Figure 6E**). Gene expression of fibrosis markers collagen (Col3a), fibronectin (Fn1) and Timp1 were elevated upon repeated low dose DT administration (**Figure 6F**). In contrast, gene expression of interferon lamba 2 (Ifnl2), also known as IL-28a, was strongly dampened (**Figure 6G**), suggesting a dysregulated lung barrier (16). Interestingly, we noticed increased expression in lung tissue of the stimulator of interferon genes (STING) protein, a major component of self-DNA sensing pathway (**Figures 6H, I** **)**, which opens further investigations towards the mechanism of immune activation and fibrosis following DT-mediated AT1 cell death.

## Discussion

A major contribution of the alveolar epithelium in the development of chronic lung diseases including COPD and ILD such as IPF is established (17). Cellular senescence may be a contributory mechanism in chronic lung diseases (18). Gradual loss of alveolar structure and cellular senescence have been reported in pulmonary emphysematous lesions of COPD patients (19, 20). IPF was long considered to be a chronic inflammatory disorder, which gradually progresses to established fibrosis. However, the failure of multiple anti-inflammatory therapies led to a paradigm change (9, 21).

The current hypothesis gives a central role to recurrent local micro-injuries to ageing alveolar epithelium which activate fibroblast proliferation and extracellular matrix deposition (2), a theory initiated a long time ago by Wanda Haschek and Hanspeter Witschi (22). A pivotal contribution of alveolar epithelial cells in IPF has been clearly identified, characterized by a loss of AT1 cells and dysfunctional AT2 cells (5). AT2 cells from IPF patients display impaired self-renewal capacity, consistent with AT2 stem-cell failure and AT1 cell loss (23).

Here, we report that airway administration of lose dose (1 ng) DT in iDTR^fl/fl^ Aqp5^CRE^ mice causes AT1 cell death associated with apoptosis as shown by increased cleaved caspase-3 staining and disrupted alveolar wall integrity. This led to increased inflammatory cell recruitment, mostly neutrophils, and early lung inflammation in iDTR^fl/fl^ Aqp5^CRE^ mice, but not in control iDTR^fl/fl^ Aqp5^WT^ mice.

Higher DT dose (10 ng) for three days led to a significant loss of alveolar wall integrity in iDTR^fl/fl^ Aqp5^CRE^ mice but not in control iDTR^fl/fl^ Aqp5^WT^ mice as evidenced by elevated numbers of Pdpn^+^ cells recovered from the alveolar lavage at day eight. This was accompanied by significant body weight loss in the iDTR^fl/fl^ Aqp5^CRE^ mice starting three days after the last DT administration. Interestingly, at this time point there was a marked airway eosinophilia while in the lungs we noticed a significant increase of both neutrophils and macrophage subsets. Markers of tissue remodeling were also elevated in the lungs together with collagen levels. Histological analyses of lung sections revealed increased inflammation and injury in the DT-treated iDTR^fl/fl^ Aqp5^CRE^ mice as compared to their iDTR^fl/fl^ Aqp5^WT^ littermates.

We then wanted to use a milder protocol with low dose DT to investigate the response at a later time point. Two DT treatments (1 ng) induced a moderate and transient weight loss in the iDTR^fl/fl^ Aqp5^CRE^ group starting three days after the last DT administration, with a full recovery by day twelve. Interestingly, AT1 cells were still recovered from the BAL, however in low numbers as compared to the previous protocol, together with low grade recruitment of both innate and adaptive immune cells. Lung histology and gene expression analyses showed increased inflammation and interstitial fibrosis in DT-treated iDTR^fl/fl^ Aqp5^CRE^ mice as compared to their iDTR^fl/fl^ Aqp5^WT^ littermates. Interestingly, Ifnl2 (IL-28A) gene expression was strongly suppressed in DT-treated iDTR^fl/fl^ Aqp5^CRE^ mice. IL-28, often regarded as a mucosal protective cytokine mainly produced by epithelial cells and dendritic cells and signaling primarily on epithelial cells (16, 24), appears strongly downregulated upon AT1 cell ablation, which may suggest dysregulated antiviral immune responses. We also observed increased STING expression, a major innate sensor involved in self-DNA sensing (25). Both IL-28 and STING results will be pursued in follow-up investigations. We previously addressed the role of STING-dependent self-DNA sensing in other models of lung inflammation and fibrosis (15, 26–28). We also previously generated mice with AT1 cell-specific deletion of MyD88 using Aqp5^CRE^, which showed a major reduction of papain-induced acute lung inflammation (29).

While we show here that DT treatment induces apoptotic cell death, we did not investigate in detail the mechanisms involved. DT induces cell stress that may activate multiple pathways, among which is endoplasmic reticulum (ER) stress, which may contribute to pulmonary fibrosis. AT2 cell-specific loss of the ER stress regulator glucose regulated protein 78 (grp78) cells causes apoptosis, senescence together with upregulation of TGF-β/SMAD signaling leading to fibrosis, similarly to what occurs in aged mice and IPF patients (30). Further studies are necessary to characterize the exact mechanisms of DT-mediated cell stress/death as well as potential subsequent AT2 cell proliferation and differentiation into AT1 cells (10, 31). In addition, it is also possible that DT-triggered AT1 cell death induces remodeling of remaining AT1 cells in a non-proliferative mechanism as well as AT1 proliferation to some extend (32, 33). Furthermore, it was recently shown that AT1 cells consists of two distinct subtypes that that differ in their cell fate (34).

Several reports analyzed the effects of targeted AT2 cell ablation. A transgenic mouse strain was generated to express the human DTR in AT2 cells driven by the surfactant protein C (SPC) promoter. Upon daily DT intraperitoneal injection for 14 days, a twofold increase in lung hydroxyproline content was found and histological evaluation revealed diffuse collagen deposition suggesting mild fibrosis (35). Another mouse model used the tamoxifen-inducible SPC-creER^T2^ system to drive DT-triggered cell death in SPC-expressing AT2 cells to show that AT2 cells display stem cell activity in adult lung (36). While extensive AT2 cell death was observed, no substantial lung injury or fibrosis were reported (36). Another transgenic mouse model was created, in which AT2 cell-specific SPC promoter drives the expression of a mutant SR39TK herpes simplex virus-1 thymidine kinase inducing dose-dependent AT2 cell depletion upon ganciclovir administration (37). The authors show that AT2 depletion leads to increased lung collagen content and fibrotic foci (37), hypothetically due to a decreased pool of AT2 precursor cells to generate AT1 cells.

To our knowledge, no AT1 cell ablation model has been reported so far. This is especially relevant since AT2 cells are AT1 progenitors upon injury and thus highly interconnected. Here, AT1 cell-specific transgenic mice were generated by inserting a CreIRES-DsRed cassette into exon 1 of the endogenous aquaporin 5 (Aqp5) gene and crossing these mice with iDTR flox mice. AQP5-driven CRE expression in AT1 cells enables the removal of a loxP-floxed STOP cassette and thus DTR expression which lead to cell death upon DT treatment. While in rat lungs AQP5 appears to be expressed exclusively on the apical membrane of AT1 and not AT2 cells (38, 39), we cannot rule out that some AT2 cells were also ablated in our system. In the original article reporting a directed CRE expression in AT1 cells using the *Aqp5* gene, it was reported that in C57BL/6J background low levels of AQP5 expression were also found in AT2 cells (13). Of note, measurable levels of AQP5 expression were detected in other organs such as submandibular salivary gland, trachea and stomach (13). These mice were further utilized to show that AT1 cells display a major role in alveolar fluid clearance (40).

In conclusion, our data demonstrate that repeated local DT administration in iDTR^fl/fl^ Aqp5^CRE^ mice leads to AT1 cell death followed by inflammation leading to tissue remodeling, defective repair and interstitial fibrosis. Therefore, this a new genetic model of controlled repeated AT1 cell death driving inflammation and interstitial pulmonary fibrosis which may be of interest to investigate further the mechanisms, progression the fibrotic process and to test drug candidates.

## Data availability statement

The original contributions presented in the study are included in the article/**Supplementary Materials**. Further inquiries can be directed to the corresponding authors.

## Ethics statement

The animal studies were approved by French Government animal experiment regulations under APAFIS#21753. The studies were conducted in accordance with the local legislation and institutional requirements. Written informed consent was obtained from the owners for the participation of their animals in this study.

## Author contributions

SC: Formal Analysis, Investigation, Methodology, Writing – review & editing. DDR: Investigation, Methodology, Writing – review & editing. DG: Formal Analysis, Methodology, Writing – review & editing. EC: Investigation, Methodology, Writing – review & editing. SH-M: Investigation, Methodology, Writing – review & editing. FS: Investigation, Methodology, Writing – review & editing. EK: Investigation, Methodology, Writing – review & editing. VQ: Funding acquisition, Writing – review & editing. AG: Investigation, Methodology, Writing – review & editing. IC: Writing – review & editing. BR: Conceptualization, Project administration, Resources, Writing – original draft, Writing – review & editing. MLB: Conceptualization, Methodology, Resources, Writing – review & editing. NR: Conceptualization, Formal Analysis, Methodology, Project administration, Supervision, Validation, Writing – original draft, Writing – review & editing.
