# Supplementary material for: Lung inflammation and interstitial fibrosis by targeted alveolar epithelial type I cell death
Source: Front Immunol. 2023 Sep 28;14:1261483. doi: 10.3389/fimmu.2023.1261483 (PMC10568624; doi:10.3389/fimmu.2023.1261483)
Supplement: Supplementary file 1 [file Table_1.pdf]

Supplementary Table 1

Target	Fluorophore	Provider
B220	BV711	BioLegend
CD103	BV780	BD Horizon
CD11b	BV421	eBioscience
CD11b	PE	BD Pharmigen
CD11c	BV605	BioLegend
CD24	BV650	BD Biosciences
CD3	AF700	eBioscience
CD4	BV650	BioLegend
CD4	Fitc	eBioscience
CD45	BV785	BioLegend
CD45.2	AF700	eBioscience
CD45.2	PerCP-Cy5.5	eBioscience
CD8a	PerCP-Cy5.5	eBioscience
CD8b	eF450	eBioscience
F4/80	PE-Cy7	eBioscience
F4/80	PE	eBioscience
IA/IE	BV711	BioLegend
Ly-6C	PerCP-Cy5.5	eBioscience
Ly-6G	FITC	BioLegend
PanCK	APC	Invitrogen
Siglec F	PE-Cf594	BD Biosciences
Siglec-F	PE	BD Pharmigen
TCRγδ	APC	eBioscience
Podoplanin	BV421	BioLegend
Podoplanin	-	ab256559
Prosurfactant protein C	-	Sigma
Cleaved caspase 3	-	Cell Signaling
β-actin	-	Sigma
STING	-	Cell Signaling

Cat#	Clone	Application
103255	RA3-6B2	FC
564322	M290	FC
48-0112-82	M1/70	FC
557397	M1/70	FC
117334	N418	FC
563545	M1/69	FC
56-0032-82	17A2	FC
100546	RM4-5	FC
11-0042-85	RM4-5	FC
103149	30-F11	FC
56-0454-82	104	FC
45-0454-82	104	FC
45-0081-82	53-6.7	FC
48-0083-82	H35-17.2	FC
25-4801-82	BM8	FC
12-4801-82	BM8	FC
107643	M5/114.15.2	FC
45-5932-82	HK1.4	FC
127606	1A8	FC
MA1-10325	AE1 & AE3	FC
562757	E50-2440	FC
552126	E50-2440	FC
17-5711-82	eBioGL3	FC
127423	8.1.1	FC
Abcam	8.1.1	IF
AB3786	Polycolonal	IF
9661S	Polycolonal	IF
A3854	AC-15	WB
13647S	D2P2F	WB
